# The Common Task

**Published:** 1907-04-27

**Authors:** 


### THE COMMON TASK.

**Correspondence and Queries** for this section should be sent to the Editor of The Hospital, 28 Southampton Street, Strand, London, and marked "Nursing Administration."

### The Care of Milk in Institutions.

In an ideal hospital system the milk is watched over during the whole of its transit from the cow to the patient. In provincial hospitals it would be perfectly feasible in most districts to follow the admirable plan at Addenbrooke's, Cambridge, where the whole milk supply is derived from one farm in the neighbourhood, which is open to inspection by the authorities. But in London, in the existing state of the milk trade, this cannot be managed. The concern of the hospital with the supply begins at the delivery of the milk on the hospital premises by the contractor. The practice of sterilising the milk on its arrival is now almost universal. When this is done the milk is delivered direct into the kitchen, and the first process, that of measuring it, is gone through. The quantity received is carefully checked by the steward or housekeeper, and a specimen is drawn off to be tested by a lactometer, that the proportion of cream may be ascertained. In most contracts for milk a proviso is inserted that the proportion of cream shall not fall below a stated figure. As it is quite easy to adulterate milk in such a way that the percentage of cream is artificially raised, it is essential that from time to time the milk should be analysed, and every month or two a specimen should be drawn off on arrival, sometimes from the morning, sometimes from the afternoon, milk, and sealed up for analysis by someone in authority. After measuring, the milk is poured straight into the steriliser.

It may be thought superfluous to dwell upon the importance of using for sterilisation a vessel kept exclusively for this purpose. Yet in a large institution the writer saw quite lately large boilers used promiscuously for the making of soup, the boiling of vegetables, and the sterilisation of milk. Such a practice, to say the least of it, throws an undue strain on the vigilance of the kitchen staff. The Benham steriliser in use at University College Hospital, where something like 40 gallons of milk are daily dealt with, is excellent in design; it consists of a copper inner pan, galvanised wrought iron jacket, and water jacket between jacket and pan, fitted with copper steam coil and water gauge. The price of the 30-gallon size is £31. An indicator is attached to the thermometer within, and it thus requires the smallest amount of attention by the cook. The "scum" which gathers on the surface is carefully strained, so that the cream is as far as possible returned to the milk, but there must always be—and this is one of the drawbacks to sterilisation—a certain proportion of "scum" left. The cook can, however, turn this to very good account, and a pint of good clotted cream may be expected to result from every 20 gallons of sterilised milk. When the milk has been ten minutes at a temperature of from 200 to 210°, it is allowed to cool off, and is then poured into covered cans for despatch to the wards, the quantity required in each ward being carefully measured out in conformity with the requisition sheet. It is stored in the ward kitchens in these covered cans, and is thence issued direct to the patients.

Where sterilisation is not practised, the dairy or larder in which the milk is received and stored is of the first importance. A cool aspect, good ventilation, a stone floor, and clean whitewashed walls, will constitute a larder in which no trouble is likely to arise with the milk, but if slate slabs and white tiling can be had a better appearance can be made. In no case should any article except milk and butter be found therein. The difficulty begins when the supply is despatched to the ward kitchens, where, under adverse atmospheric conditions, unboiled milk will need very careful watching. In some hospitals the milk is sterilised in the ward kitchens. Comparatively simple apparatus is required for this purpose, merely an ordinary milk warmer, consisting of an urn lined with earthenware jar and fitted with "water jacket," but taken collectively the time spent over the process in the various wards is considerable, and constitutes a certain waste of labour. Inaccuracy in measuring is responsible for most of the waste which takes place in the milk supply in institutions, and it may be taken as an axiom that waste of this article of consumption is more usual in small hospitals than in large ones; for precautions which are taken as a matter of course over an amount of 50 gallons or so, are often omitted altogether over five.

Nearly everybody is agreed that sterilisation is merely a necessary evil pending the purification of the milk supply, and the question is inextricably connected with the problem why there is always a little black sediment at the bottom of a glass of milk. From a thoroughly hygienic dairy, milk properly cooled and conveyed in clean vessels, and uncontaminated at any point in its travels, remains sweet for three days at the least. Why should not this be always the case?

